# Acknowledgment to the Reviewers of *Medical Sciences* in 2022

**DOI:** 10.3390/medsci11010010

**Published:** 2023-01-17

**Authors:** Medical Sciences Editorial Office

**Affiliations:** MDPI AG, St. Alban-Anlage 66, 4052 Basel, Switzerland

High-quality academic publishing is built on rigorous peer review. *Medical Sciences* was able to uphold its high standards for published papers due to the outstanding efforts of our reviewers. Thanks to the efforts of our reviewers in 2022, the median time to first decision was 20 days and the median time to publication was 47 days. Regardless of whether the articles they examined were ultimately published, the editors would like to express their appreciation and thank the following reviewers for the time and dedication that they have shown *Medical Sciences*:

Abul Kalam AzadLee Bulla, Jr.Adina FésüsLinda MadsenAgata GóźdźLisa ShantzAgata JanowskaLuca Di GianfrancescoAikaterini D. KoutsouLuca GiacomelliAkkan AvciMª Àngels ColomerAlessandro GennaiMagdalena KołodziejAlessandro MalobertiMałgorzata BiałekAlessandro MeduriManuela A. HoffmannAlessandro PoggiMaralyn FoureurAlexandre SilvaMarcus ScottiAlexandru Florin RogobeteMaria Luisa Di PaoloAlfredo Téllez-ValenciaMaria MazzitelliAlvaro MaciasMariusz KlenckiAmiya PatraMariya PakharukovaAmy Tu WangMarta LaranjoAnastasios ApostolosMartín Pérez-SantosAndrea IlariMasataka SumiyoshiAndreia Martins RosaMaurice MarsAndreu Comas-GarciaMegan CrichtonAng LiMelania BalasAngelos SikalidisMichael Friedrich BoettcherAnisuz ZamanMichel PérusseAnna BaranMiguel Angel OrtegaAnna BläckbergMika Kondo KuniedaAnthony PappMiljana JovandaricAntonio MaccioMiran RadaAntonio MassaroMohammad FarhanArda GülerMonika Łopuszańska-DawidArif SiddiquiMonika Musiał-KulikAshok KumarMuhammad Mubashar IdreesAthira N. SurendranMyriam RichaudAtiđa SelmaniMythili DileepanAtsushi KohgaNicola PalenaAtsushi SanoNimrat ChatterjeeAušra MongirdienėNitikaBabak PakbinOliver SommerfeldBeata Hukowska-SzematowiczOndrej KrejcarBidisha MitraPankaj SharmaByung Gun LimPaul SchoenhagenCarlo PerisanoPernille HolmagerCarlos GasparPiyanuch KongtimCarlos Ruiz-NuñezPrasenjit SahaCarmine NovielloQin Xiang NgCaroline SubraRafał MańczakCarsten CarlbergRaimund WinterCassandra E. HolbertRaúl Carrera CerritosCelina WojciechowskaRavi Prakash SahuChan Hee ParkReinhard ZelenyChristina SakarikouRobert Yung-Liang WangCiro ManzoRocco Salvatore CalabròClaudia KusmicRolf TeschkeCoralie L'OllivierSadanand PandeyCorina S. PopSaed KhaliliehDaniel SteinmannSaid BoumarafDaniela AraújoSang Hun KimDaniela CalinaSanthosh MukundanDanuta Gąsior-PerczakSastre-Garau XavierDavid F. KisorSavvas LampridisDebjani PalSebastian WillenborgDianjun CaoSerguei Alejandro-MartínDon BenjaminShih-Yen LoDong-Jin HwangShravan GiradaElena BernadShusuke TodenElisabetta BertelliniShweta KukrejaErika CioneSibaji SarkarFaisal RazaSiddharth PahwaFarid RahimiSimone BeninatiFatemeh TabatabaieSoichiro TakamiyaFilip DabrowskiSoumaya KouidhiFilippo ZilioStefan HadzicGabriel Claudiu TenderStefania HanauGabriele MessinaStefania MaiGeorge PadosStefano TavanoGiada MondanelliStergios BoussiosGiampaolo VettaSung-Ho LeeGiuseppe MasciaSvjetlana RausGuglielmo ManticaTabito KinoGustavo ArocaTakeshi UemuraHafize Emine SönmezTarek MohamedHan-Mou TsaiTatsiana PukhalskayaHanna LiberskaTerrence J. RavineHans BrunnströmTin PhanHui YeTingyu ChangIffath Unissa SyedTorsten PastorIgor Borisowitsch BuchwalowVasiliki TsigkouIlia BeberashviliVeronica VelascoIván CarreraVincenzo Giannicola MendittoJ. Michael SorrellVishakha Anand PawarJaishankar BharatharajWayne CarverJan SteenekampWei-Chieh LeeJaneen R. JordanXiaohua WuJooho ParkXiaoming WangJorge RodriguesXin ZhangJuan Barges-CollXingsi XueJurema Corrêa Da MotaYajuan SuKamonwon IenghongYi HuangKarel SmetanaYuening LiuKathryn LeyvaYu-Hsun KaoKatyayani TatipartiYuichi TsuchiyaKe-Vin ChangYukio MaruyamaKevin FiteZhengnian LiKevin MeestersZhengui YanKumiko SaekiZhiping LiuLaurie BruzzeseZhuoran Ma

